# NanoBRET: The Bright Future of Proximity-Based Assays

**DOI:** 10.3389/fbioe.2019.00056

**Published:** 2019-03-26

**Authors:** Natasha C. Dale, Elizabeth K. M. Johnstone, Carl W. White, Kevin D. G. Pfleger

**Affiliations:** ^1^Molecular Endocrinology and Pharmacology, Harry Perkins Institute of Medical Research, QEII Medical Centre, Nedlands, WA, Australia; ^2^Centre for Medical Research, The University of Western Australia, Crawley, WA, Australia; ^3^Australian Research Council Centre for Personalised Therapeutics TechnologiesAustralia; ^4^Dimerix Limited, Nedlands, WA, Australia

**Keywords:** BRET, NanoLuc, Nluc, NanoBRET, fluorophore, ligand binding, CRISPR

## Abstract

Bioluminescence resonance energy transfer (BRET) is a biophysical technique used to monitor proximity within live cells. BRET exploits the naturally occurring phenomenon of dipole-dipole energy transfer from a donor enzyme (luciferase) to an acceptor fluorophore following enzyme-mediated oxidation of a substrate. This results in production of a quantifiable signal that denotes proximity between proteins and/or molecules tagged with complementary luciferase and fluorophore partners. BRET assays have been used to observe an array of biological functions including ligand binding, intracellular signaling, receptor-receptor proximity, and receptor trafficking, however, BRET assays can theoretically be used to monitor the proximity of any protein or molecule for which appropriate fusion constructs and/or fluorophore conjugates can be produced. Over the years, new luciferases and approaches have been developed that have increased the potential applications for BRET assays. In particular, the development of the small, bright and stable Nanoluciferase (NanoLuc; Nluc) and its use in NanoBRET has vastly broadened the potential applications of BRET assays. These advances have exciting potential to produce new experimental methods to monitor protein-protein interactions (PPIs), protein-ligand interactions, and/or molecular proximity. In addition to NanoBRET, Nluc has also been exploited to produce NanoBiT technology, which further broadens the scope of BRET to monitor biological function when NanoBiT is combined with an acceptor. BRET has proved to be a powerful tool for monitoring proximity and interaction, and these recent advances further strengthen its utility for a range of applications.

## Introduction

A number of widely used technologies to study cellular biology are reliant upon luciferase enzymes isolated or derived from organisms that produce bioluminescence. These include luciferases derived from the click beetle and the North American firefly (Fluc), both of which catalyze the ATP-dependent metabolism of D-luciferin to produce luminescence (Wood et al., 1989; Fraga, 2008; England et al., 2016), as well as luciferases with ATP-independent metabolic reactions, such as the sea pansy-derived *Renilla* luciferase (Rluc) (Lorenz et al., 1996).

Rluc and the mutated derivative Rluc8 (Kocan et al., 2008) have been widely used for bioluminescence resonance energy transfer (BRET), a biophysical technique to monitor proximity within live cells. BRET has been used extensively in pharmacological research, particularly in relation to G protein-coupled receptors (GPCRs) (Pfleger and Eidne, 2005; Lohse et al., 2012).

The latest addition to the luciferase toolkit is the small (19 kDa) luciferase subunit Nanoluciferase (NanoLuc; Nluc) derived from a larger multi-component luciferase isolated from the deep sea shrimp *Oplophorus gracilirostris* (Hall et al., 2012). In conjunction with its complementary substrate furimazine, Nluc's small size and superior luminescence profile has led to its rapid uptake in research, replacing other luciferases where increased sensitivity is required, while also leading to the development of new experimental approaches. Its use as a luciferase in BRET assays has resulted in the creation of the new BRET methodology termed NanoBRET (Machleidt et al., 2015; Stoddart et al., 2015).

Comprehensive reviews and protocols of the traditional BRET methodologies including their uses and variations have been published previously (Milligan, 2004; Hamdan et al., 2006; Pfleger and Eidne, 2006; Pfleger et al., 2006b; Prinz et al., 2006; Lohse et al., 2012), and as such this review will not discuss these approaches in detail. Instead, this review will focus on Nluc and the advantages and novel uses of NanoBRET and other related Nluc-based assays.

## Bioluminescence Resonance Energy Transfer (BRET)

**Figure 1:**
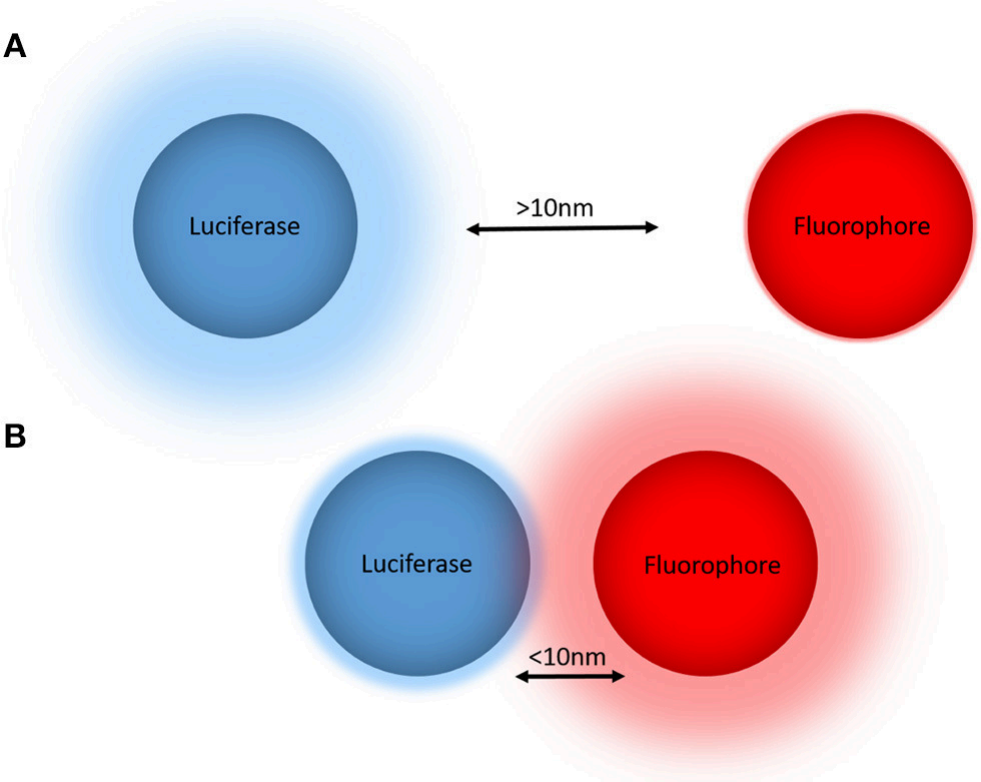
The principle of bioluminescence resonance energy transfer (BRET) for monitoring biological proximity. **(A)** The donor luciferase and acceptor fluorophore are not in close proximity (>10 nm), such that no resonance energy transfer occurs and there is no emission from the fluorophore. **(B)** The donor luciferase and acceptor fluorophore are in close proximity (<10 nm), allowing BRET to occur that reduces the donor light emission and results in light emission from the acceptor. When these BRET tags are fused to proteins or small molecules of interest, the non-radiative energy transfer from the donor luciferase to the acceptor fluorophore produces a change in the BRET ratio that in turn indicates proximity of the tagged proteins and/or small molecules.

BRET is a biophysical technique used to study proximity within live cells (Pfleger and Eidne, 2006). It relies on the naturally occurring process of dipole-dipole non-radiative energy transfer from a luciferase energy donor to an acceptor fluorophore following oxidation of a luciferase substrate. As energy transfer occurs only when the donor and acceptor are within close proximity (<10 nm) (Wu and Brand, 1994; Dacres et al., 2012), attaching the donor and acceptor tags to proteins of interest allows for protein-protein proximity to be monitored in a highly specific manner (Pfleger and Eidne, 2006) (Figure 1). Furthermore, demonstration that the BRET approach also works very effectively when a small acceptor fluorophore such as boron-dipyrromethene (BODIPY) is conjugated to a small molecule (Stoddart et al., 2015) has extended its applicability substantially.

The BRET methodology first appeared in the literature when Xu et al. (1999) used it as a method to investigate interactions of proteins involved in circadian rhythms within cyanobacteria. This initial BRET methodology used Rluc as the energy donor with its substrate coelenterazine and an enhanced yellow fluorescent protein (EYFP) as the energy acceptor. However, Xu et al. (1999) noted that the method could be extrapolated to the use of any appropriate luciferase-fluorescent protein combination. There are three major requirements to conduct a BRET assay: (1) proteins/molecules of interest must be labeled with the appropriate donor luciferase and acceptor molecule in a manner that does not unduly compromise their function; (2) tagged proteins/molecules must be configured and localized appropriately within the desired experimental environment; and (3) access to an appropriate instrument to monitor energy transfer is needed, most often a microplate reader that can measure in real-time using live cells (Pfleger and Eidne, 2006; Stoddart et al., 2016).

Following the study by Xu et al. (1999), multiple variations of BRET have subsequently been developed (Table 1). BRET^2^ uses coelenterazine 400a (also known as DeepBlueC^TM^), which shifts the emission peak of Rluc from about 475 to 480 nm with coelenterazine h—the coelenterazine derivative most commonly used to conduct BRET^1^–to about 395–400 nm (Pfleger and Eidne, 2006). It also typically uses green fluorescent protein (GFP) derivatives GFP^2^ or GFP10 as the energy acceptor. BRET^2^ provides an alternative to the initially developed coelenterazine h-based BRET, now often referred to as BRET^1^, with BRET^2^ producing greater spectral separation between the donor and acceptor emission peaks resulting in lower background signal. However, the utility of coelenterazine 400a is limited by its rapid decay kinetics and very low luminescence output (Hamdan et al., 2005; Pfleger and Eidne, 2006). Variations of the BRET method have further broadened its experimental scope to include assays monitoring protein-protein interaction (PPI) kinetics over extended time-scales (Pfleger et al., 2006a), including temporal monitoring of receptor trafficking through subcellular compartments (Lan et al., 2012; Tiulpakov et al., 2016).

**Table 1:** Comparison of various BRET techniques.

	**BRET^**1**^**	**BRET^**2**^**	**eBRET**	**NanoBRET**	**eNanoBRET**
Luciferase	Renilla Luciferase (Rluc/Rluc8) (36 kDa)	Renilla Luciferase (Rluc/Rluc8) (36 kDa)	Renilla Luciferase (Rluc/Rluc8) (36 kDa)	Nanoluciferase (Nluc) (19 kDa)	Nanoluciferase (Nluc) (19 kDa)
Approximate luciferase emission peak	475–480 nm	395–400 nm	475–480 nm	~460 nm	~460 nm
Substrate	Coelenterazine h	Coelenterazine 400a	EnduRen	Furimazine	Endurazine (Vivazine)
Common energy acceptors and approximate emission peak	YFP Venus (527 nm)	GFP10 GFP^2^ (~510 nm)	YFP Venus (527 nm)	HT-NCT (635 nm) Venus (527 nm) BODIPY (variable) TAMRA (~579 nm)	HT-NCT (635 nm) Venus (527 nm) BODIPY (variable) TAMRA (~579 nm)
Potential assay duration	Approximately 1 h	Seconds	>6 h	Approximately 2 h	>6 h
Advantages	Widely used and well-established technique in standard overexpression systems	Greater emission peak separation leading to decreased background signal	Similar to BRET^1^, but allows proximity to be monitored for extended time periods	Improved sensitivity and various novel applications enabled, including ligand binding and BRET using genome-edited proteins	Allows NanoBRET assays to be conducted over extended time periods
Limitations	Does not appear to be amenable to binding studies requiring extracellular luciferase-tagging of receptor. Poor sensitivity for BRET when using genome-edited proteins.	Same as for BRET^1^, plus substantially lower luminescence output and rapid substrate decay largely limits current use to intracellular biosensors involving intramolecular BRET	Same as for BRET^1^, plus requirement for substrate pre-incubation period	High luminescence output can saturate detector—reduced detector gain or delay in reading may be required.	Requirement for substrate pre-incubation period

## Nanoluciferase (NanoLuc; Nluc) and NanoBRET

### Nluc

The most recently developed commercially-available luciferase enzyme is the small (19 kDa) luciferase subunit Nluc. Nluc was initially derived from the naturally occurring luciferase present in the deep sea shrimp *O. gracilirostris* and has been optimized to produce a luciferase enzyme subunit with improved luminescence and stability (Hall et al., 2012).

The naturally occurring heteromeric luciferase produced by *O. gracilirostris* comprises two 35 kDa subunits and two 19 kDa subunits. The bioluminescent properties of the enzyme were discovered to be the result of the smaller 19 kDa subunits (Inouye et al., 2000). Hall et al. (2012) investigated this subunit and the complementary coelenterazine substrate for structural optimization, conducting three major iterations of optimization. Random mutagenesis of the 19 kDa subunit was performed, followed by screening of the products for increased luminescence. Eight mutations that improved luminescence were identified and used to produce the enzyme subunit variant upon which coelenterazine analogs were tested. Twenty-four coelenterazine analogs were screened and the analog that produced the greatest luminescence, 2-furanylmethyl-deoxy-coelenterazine, was subsequently given the name furimazine. Finally, a second group of beneficial mutations in the luciferase subunit were identified when screened with furimazine. These subsequent mutations were integrated into the previously produced variant to produce Nluc. The net result of these optimizations is greatly increased luminescence (~150x that of Fluc or Rluc) and stability (half-life >2 h) when compared to the wild-type 19 kDa luciferase, as well as luciferases such as Rluc and Fluc (Hall et al., 2012).

Nluc was initially tested for its viability as a genetic and fusion reporter (Hall et al., 2012). As a genetic reporter, Hall et al. (2012) demonstrated that Nluc can be successfully inserted into expression plasmids and used to measure changes in gene expression, producing the same results as previous methods utilizing Fluc. Following on from this initial study, Nluc has been utilized as a genetic reporter in multiple studies (Heise et al., 2013; Hikiji et al., 2015; Masser et al., 2016). Other fusion constructs were also produced and used for bioluminescent imaging, further highlighting Nluc's validity as a fusion reporter, while also demonstrating Nluc's novel application for imaging studies, an application that was limited with previous luciferase reporters but is enabled by Nluc's enhanced luminescence and stability profile (Hall et al., 2012).

Following its development, Nluc has been used for a multitude of applications including the investigation of PPIs through NanoBRET and Nluc Binary Technology (NanoBiT), gene regulation, protein stability and imaging (England et al., 2016). Nluc has also shown utility beyond the realm of classical exogenous *in vitro* experimentation with Nluc successfully used for *in vivo* bioluminescent imaging (Stacer et al., 2013; Karlsson et al., 2015; Alcobia et al., 2018) as well as the tagging of biological agents such as viruses (Karlsson et al., 2015; Anindita et al., 2016) and bacteriophages (Zhang et al., 2016), bacteria (Loh and Proft, 2014) and parasites (Azevedo et al., 2014; De Niz et al., 2016). Nluc's greater luminescence and stability, along with its smaller size, results in it being a highly versatile and powerful tool to interrogate molecular function.

### NanoBRET

Until recently, BRET assays predominantly used a combination of an ATP-independent luciferase (e.g., Rluc8) as the energy donor, a GFP variant (e.g., Venus) as the energy acceptor and coelenterazine h (or coelenterazine 400a for BRET^2^) as the luciferase substrate (Pfleger and Eidne, 2006). The optimization of the new luciferase reporter Nluc, as well as its substrate furimazine (Hall et al., 2012), has resulted in the development of NanoBRET, which offers advantages over previous BRET methods as well as unique opportunities for new applications of BRET assays.

Relative to Rluc with coelenterazine h, Nluc with furimazine exhibits ~150 times greater luminescence with a signal that is slightly blue-shifted (about 20 nm), giving an emission peak of about 460 nm and a spectral range ~20% narrower. Nluc also exhibits physical stability in a range of environmental conditions (Hall et al., 2012). Nluc has two major properties that make it advantageous over previous BRET luciferases. Firstly, Nluc's increased luminescence allows for novel BRET assays to be developed to utilize the increased assay sensitivity. For example, allowing NanoBRET to monitor low prevalence PPIs that may not be detectable with traditional BRET assays, as well as allowing for monitoring of PPIs at lower and oftentimes more physiologically-relevant levels (Mo et al., 2016). Additionally, even though the spectrum of Nluc is left shifted from Rluc, its intense brightness results in substantial emission at longer wavelengths. This allows energy transfer to occur with a broad range of colored fluorophores, enabling improved spectral separation between donor and acceptor emissions. In addition to its increased luminescence, Nluc's small size relative to other luciferases also offers novel opportunities for its use in BRET. When used as a fusion reporter, Nluc is theoretically less likely to cause function-altering steric hindrance. This enables its use in BRET assays in which the possibility of altered function of the fusion construct may be of concern. Furthermore, its small size and capacity to fold appropriately in extracellular environments has enabled its use in receptor-ligand binding studies where Nluc is fused to the extracellular N′-terminus of the receptor without impairing localization at the plasma membrane (Stoddart et al., 2015).

Multiple studies have now demonstrated Nluc's capabilities as an energy donor for use in NanoBRET experiments (Machleidt et al., 2015; Robers et al., 2015; Shigeto et al., 2015; Stoddart et al., 2015; Boute et al., 2016; Mo et al., 2016; Eyre et al., 2017; Moreno et al., 2018; Wan et al., 2018; Hoare et al., 2019; White et al., 2019). For example, Eyre et al. (2017) utilized Nluc and the red fluorescent protein mKate2 to conduct NanoBRET experiments to monitor the conformation and dimerization of NS5A, a protein vital to essential functions of the hepatitis C virus such as RNA replication, and therefore a major target for anti-hepatitis C therapeutics. Furthermore, a study by Robers et al. (2015) describes a NanoBRET protocol for monitoring small molecule-binding to an intracellular target using an Nluc-tagged intracellular protein of interest and a cell-permeable fluorescent dye-ligand conjugate. Using this method small molecule binding to an intracellular target was monitored through displacement of the fluorescent ligand. A similar method was subsequently used to investigate the temporal aspects of small molecule engagement with the intracellular target. This technique has also been used successfully to investigate the binding of kinase inhibitors to a panel of kinases and the subsequent effect the presence of cellular ATP has upon the binding of various kinase inhibitors (Vasta et al., 2018). These studies demonstrate the possibility of using NanoBRET to investigate small molecule pharmacology directly at intracellular targets, with exciting implications for the interrogation of intracellular-acting small molecules (Robers et al., 2015; Vasta et al., 2018).

The increased luminescence of Nluc has additionally enabled NanoBRET to be used for imaging. Kim and Grailhe (2016) used a combination of Nluc and YFP to image NanoBRET using a widefield high-content microscope and Goyet et al. (2016) used a bioluminescence-dedicated inverted fluorescence microscope to detect BRET between Nluc and Venus. Goyet et al. (2016) subsequently compared results with Nluc to results using Rluc8 and Venus to highlight the advantages of using Nluc for imaging-based NanoBRET. Furthermore, Alcobia et al. (2018) used a bioluminescence microscope to detect binding of a fluorescent ligand to Nluc-tagged β_2_-adrenoceptors. Nluc's novel properties have been increasingly exploited in a broad range of applications beyond traditional BRET assays, including: novel NanoBRET-based assays such as the biosensor termed “BTeam” developed by Yoshida et al. (2016) to measure intracellular ATP concentration; a calcium ion biosensor developed by Yang et al. (2016); and a caspase activity sensor developed by den Hamer et al. (2017).

### NanoBRET Ligand Binding

**Figure 2:**
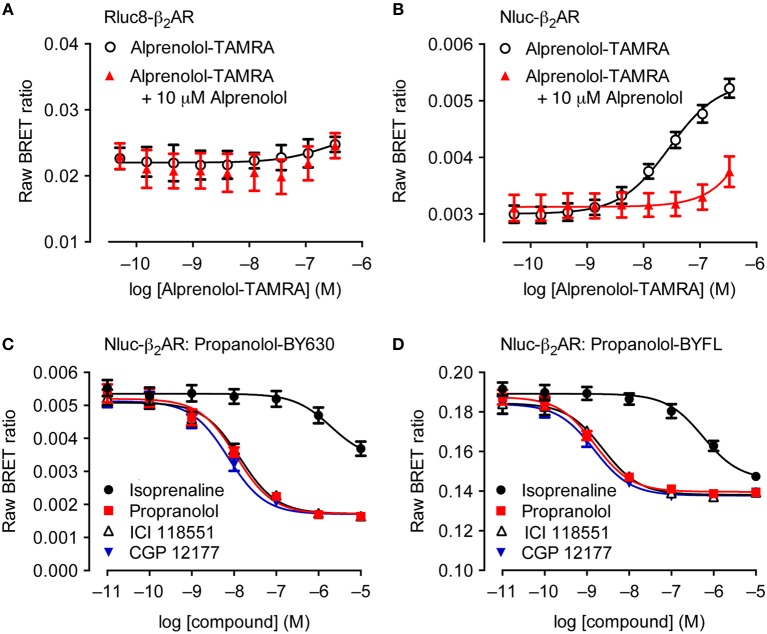
Suitability of Nluc for BRET binding studies. **(A,B)** BRET ligand binding assays for transiently-transfected Rluc8-β_2_-adrenoceptor (β_2_AR) **(A)** and Nluc-β_2_AR **(B)** treated with increasing concentrations of alprenolol-TAMRA in the absence or presence of 10 μM unlabeled alprenolol. Data are mean ± s.e.m. of three experiments performed in quadruplicate. **(C,D)** Inhibition of the BRET signal for HEK293 cells stably-expressing Nluc-β_2_AR treated with 10 nM propranolol-BY630 **(C)** or propranolol-BYFL **(D)** and increasing concentrations of unlabeled ligands as shown. Each data point represents mean ± S.E.M. of five [all curves in **(C)** and propranolol in **(D)**] or four **(D)** separate experiments. In each experiment triplicate determinations for each data point were made. Raw BRET Ratio = (long wavelength emission/short wavelength emission), data presented on log_10_ scale. Reproduced from Stoddart et al. (2015).

Although theoretically possible, the ability to study ligand binding using BRET has not been viable using traditional BRET technologies for multiple reasons. The relatively large size of luciferases such as Rluc and Fluc make extracellular N′-terminal labeling of receptors challenging due to potential steric hindrance that interferes with a ligand's ability to bind to the appropriate binding sites on the receptor (Hall et al., 2012; Stoddart et al., 2017). Additionally, it has been shown that GPCRs that are N′-terminally tagged with Rluc variants do not traffic correctly to the plasma membrane, remaining trapped within the cell, as demonstrated with luminescent imaging (Stoddart et al., 2015). This contrasts with the appropriate trafficking observed with GPCRs N′-terminally tagged with Nluc (Stoddart et al., 2015). The enhanced suitability of Nluc for extracellular tagging has in part been attributed to its derivation from a naturally secreted protein that has evolved to fold appropriately outside of the cell, as compared to intracellular luciferases such as Rluc (Liu et al., 1997). The culmination of all these advantageous properties has resulted in receptors being successfully Nluc-tagged at the extracellular N′-terminus, enabling NanoBRET to be used for monitoring ligand binding. Figure 2 illustrates that the fluorescent ligand alprenolol-tetramethylrhodamine (alprenolol-TAMRA) does not display specific binding to Rluc8-β_2_-adrenoceptor (β_2_AR) (Figure 2A), but does show specific binding to Nluc-β_2_AR (Figure 2B) that is inhibited by 10 μM unlabeled alprenolol. NanoBRET ligand binding can also be used to monitor competition binding. Figures 2C,D demonstrate competition binding curves of multiple β_2_AR antagonists with two different fluorescent ligands, propranolol-BY630 (emission peak of 650 nm; Figure 2C) and propranolol-BYFL (emission peak of 512 nm; Figure 2D), demonstrating the robustness of the assay to fluorophore choice.

NanoBRET offers an attractive alternative to traditional radioligand binding, particularly given the safety issues regarding use of radioactive isotopes. Since it was first described by Stoddart et al. (2015), the NanoBRET ligand binding assay has been used in numerous studies (Christiansen et al., 2016; Soave et al., 2016; Hansen et al., 2017; Kilpatrick et al., 2017; Wang et al., 2017; Alcobia et al., 2018; Conroy et al., 2018; Mocking et al., 2018; Peach et al., 2018; Stoddart et al., 2018; Hoare et al., 2019), including where the receptor is expressed under endogenous promotion (initiation of transcription by an endogenous promoter rather than one transfected in; White et al., 2019). Additionally, as ionizing radiation is not required for NanoBRET, this enables the characterization of low affinity ligands with micromolar concentrations. The study of such compounds is limited when using radioactive isotopes due to excessive non-specific binding as well as cost and safety limitations (Stoddart et al., 2017). Furthermore, due to the inherent proximity dependence of BRET, NanoBRET ligand binding assays produce a highly specific signal, as only ligands bound to targets tagged with Nluc will produce a signal, unlike traditional radioligand binding assays (Stoddart et al., 2015). Perhaps most importantly, the ability to investigate ligand binding kinetics in real time in live cells enabled by NanoBRET gives opportunities to study aspects of ligand binding difficult to observe with alternative techniques. Thus, the NanoBRET ligand binding assay has numerous advantages over the traditional radioligand binding assay. It is therefore likely to become a fundamental technique to study receptor-ligand interactions in pharmacology.

## Novel Fluorescent BRET Acceptors

BRET assays have traditionally employed GFP variants (e.g., YFP) as the preferred energy acceptor. Recently, the pool of fluorophore agents considered for use in BRET assays has broadened, offering exciting potential for novel BRET assay techniques and uses.

### HaloTag and NanoBRET

Along with optimization of the NanoBRET luciferase and substrate, alternative acceptor fluorophores were also considered by Machleidt et al. (2015) who utilized the HaloTag (HT) system as an alternative to GFP-based acceptors. The HT system consists of a small (33 kDa) HT protein that can be genetically fused to a protein of interest and a chloroalkane ligand that forms an irreversible covalent bond with the HT. The chloroalkane ligand can then be bound to a range of molecules, including fluorophores that can subsequently be used as acceptors for BRET assays (Los et al., 2008). Given the innate potential for this system to allow the use of multiple fluorophores, it lends itself to simple optimization of the acceptor fluorophore for individual experimental needs, as well as offering opportunities for multiplexing. Machleidt et al. (2015) optimized the use of HT with NanoBRET by choosing a red-shifted fluorophore with peak light emission at 635 nm to give maximal spectral separation from the energy donor. They found this system to produce the most favorable results, leading to decreased background signal and improved sensitivity over a range of protein concentrations when compared to Rluc8/YFP assays with coelenterazine h (Machleidt et al., 2015).

HT has subsequently been employed in numerous NanoBRET assays including: investigation of the mechanisms of activation of Rabin8, a critical endosomal recycling modulator (Wu et al., 2015); demonstration of BRET multiplexing when monitoring Nluc-tagged GPCR trafficking toward Venus-tagged Rab4 in the early endosome and away from the Kras plasma membrane marker tagged with red non-chloro TOM (NCT) ligand bound to HT in the same cells (HT-NCT; White et al., 2017); and interactions of DNA binding protein PRDM14 with glucose-regulated protein 78 (GRP78) and heat-shock protein 90-α (HSP90α) (Moriya et al., 2018). Other self-labeling protein tags e.g., SNAP or CLIP tags may readily be substituted for HT as suitable fluorescent acceptors (Hiblot et al., 2017).

### Novel Fluorescent Dyes for NanoBRET Ligand Binding

In order to monitor ligand binding with NanoBRET, appropriate fluorescent ligands are required to act as energy acceptors. Multiple studies have now demonstrated successful construction, validation, and use of fluorescent ligands to conduct NanoBRET ligand binding studies (Stoddart et al., 2015, 2018; Christiansen et al., 2016; Soave et al., 2016; Hansen et al., 2017; Kilpatrick et al., 2017; Wang et al., 2017; Alcobia et al., 2018; Conroy et al., 2018; Peach et al., 2018; Hoare et al., 2019). Three fluorescent dyes that have been used as conjugates to ligands for BRET studies are TAMRA, BODIPY, and 4-nitro-7-aminobenzofurazan (NBD).

NBD has the advantage of being small relative to the larger TAMRA and BODIPY molecules, with an excitation peak of ~470 nm and emission peak of ~530 nm. NBD also has the beneficial characteristic of exhibiting increased fluorescence in hydrophobic environments such as the plasma membrane and ligand binding pockets, similar to BODIPY discussed below (Christiansen et al., 2016; Stoddart et al., 2016). Christiansen et al. (2016) utilized this fluorophore to investigate free fatty acid receptor 1 (FFA1) agonists as potential type 2 diabetes therapeutics and theorized that NBD's specific characteristics would result in reduced background signal, which indeed was observed.

TAMRA is a commonly used fluorescent dye with an excitation peak of ~556 nm and an emission peak of ~579 nm. It is often used as a fluorescent tag for antibodies and proteins in cellular imaging, and as such the extension of its use to the tagging of ligands to monitor ligand binding is a natural progression. Stoddart et al. (2015) utilized TAMRA-tagged alprenolol and angiotensin II to observe ligand binding to Nluc-tagged β_2_AR and angiotensin AT_1_ receptors, respectively, using NanoBRET. Clear, concentration dependent changes in BRET were observed, demonstrating their utility to investigate ligand binding through this method. Kilpatrick et al. (2017) also used NanoBRET to monitor TAMRA-tagged vascular endothelial growth factor (VEGF) binding to the receptor tyrosine kinase VEGFR2. Using this ligand they also observed TAMRA-VEGF-induced internalization of Nluc-VEGFR2, followed by dissociation of TAMRA-VEGF while the receptor was internalized within endosomes.

While NBD and TAMRA have both been used successfully in NanoBRET ligand binding experiments, perhaps the most versatile fluorescent dyes are the BODIPY fluorophores. BODIPY dyes have been widely used for tagging of biological agents. They exhibit high fluorescence and absorption levels making them suitable acceptor fluorophores for use in NanoBRET ligand binding assays. The BODIPY dyes are also not limited to a single excitation/emission spectral combination, with multiple BODIPY dye variations commercially available with excitation/emission spectral ranges that span the majority of the visible light spectrum (Ziessel et al., 2007). BODIPY dyes have numerous favorable properties, including good solubility in a range of solvents, albeit not aqueous ones, and relative insensitivity to changes in polarity and pH (Ziessel et al., 2007; Gonçalves, 2008). BODIPY also lacks electrical charge and is relatively nonpolar, which minimizes any effect of the BODIPY fluorophore on the functional properties of the tagged ligand. This makes BODIPY particularly appealing for use with low molecular weight ligands (Gonçalves, 2008). Stoddart et al. (2018) utilized BODIPY630/650 to develop fluorescent histamine H_1_ receptor antagonists used to conduct NanoBRET ligand binding as well as confocal imaging, highlighting the utility and versatility of the BODIPY dyes when used as ligand conjugates.

Other commercially available fluorophores that can be used for NanoBRET ligand binding assays include the Alexa Fluor^TM^ dyes. Similarly to the BODIPY dyes, these are available with a range of excitation/emission spectra (Lichtman and Conchello, 2005) and have been frequently used in fluorescence resonance energy transfer (FRET) or time-resolved FRET studies (Albizu et al., 2010). Indeed, Nluc-SNAP or Nluc-HT chimeras generated by Hiblot et al. (2017) show tunable luciferase emission when coupled with different AlexaFluor dyes.

The high distance dependence of resonance energy transfer assays reduces the need for highly subtype selective fluorescent ligands to be developed (Hounsou et al., 2017), as binding to other receptor subtypes will not be detected if they are not tagged with Nluc unless they are in very close proximity to an Nluc tagged receptor (Jaeger et al., 2014). However, it is important to acknowledge that the addition of a fluorophore may result in changes to the properties of the ligand. Additionally, the choice of linker between the ligand and fluorophore has also been shown to alter the observed properties of the ligand (Morishima et al., 2010; Vernall et al., 2012, 2013, 2014). Vernall et al. (2013) gave an example of this by shifting the properties of a non-selective adenosine receptor antagonist to a selective antagonist with varying affinity values depending upon the alterations made to the linker. This study highlights the effect conjugation can have upon the pharmacological profile of the ligand and demonstrates the importance of understanding these consequences when conducting experiments using these compounds.

## NanoBRET With Extended Live Cell Substrates

Extended BRET (eBRET) is a derivation of BRET^1^ that uses a protected (caged) form of coelenterazine h called EnduRen^TM^, which when metabolized by intracellular esterases produces bioavailable coelenterazine h (Pfleger et al., 2006a). eBRET enables BRET assays to be conducted over extended timeframes of at least 6 h because of the equilibrium between protected substrate in the media and free coelenterazine h in the cells (Pfleger et al., 2006a). This leads to the stable and prolonged availability of coelenterazine h for oxidation by the luciferase.

Recently, extended live cell substrates have become commercially available that are optimized for use with Nluc, namely Endurazine^TM^ and Vivazine^TM^. These are protected (caged) forms of furimazine that are similarly metabolized by intracellular esterases to produce bioavailable furimazine in live cells, enabling extended NanoBRET (eNanoBRET). The Endurazine caged-furimazine has already been used as an Nluc substrate for NanoBRET to assess receptor-arrestin interactions in real time over an extended time-course, with arrestin tagged with the energy acceptor in the form of either Venus YFP or red NCT bound to HT (Tiulpakov et al., 2016). Similar to EnduRen when compared to coelenterazine h (Pfleger et al., 2006a), Endurazine results in lower initial luminescence intensity than furimazine, but once equilibrium is reached, the luminescence from Endurazine is stable for many hours. In addition, Hattori et al. (2016) developed a furimazine substrate that is uncaged by UV illumination for use in confocal bioluminescence imaging, though to date this has not been used in BRET assays.

## NanoBRET and *in vivo* Studies

Luciferases have been widely used *in vivo* as reporters (Contag et al., 1997). However, to increase luminescence output and tissue penetrance BRET reporters termed “Nano-lanterns” have also been developed. Nano-lanterns consist of a luciferase fused to a fluorescent protein and results in highly efficient intramolecular energy transfer. This not only increases the emitted photon number but such reporters can be “tuned” to particular wavelengths by the use of different fluorescent proteins (Saito et al., 2012; Rumyantsev et al., 2016). However Nluc, with its dramatically increased luminescence output over previous luciferases, enables bioluminescence methods to be utilized with greater sensitivity *in vivo*, including NanoBRET (Schaub et al., 2015; Chu et al., 2016). Similar to NanoLanterns, Schaub et al. (2015) developed fusion reporters termed “LumiFluors” of Nluc conjugated with a fluorescent protein [either enhanced GFP or long Stokes shift (LSS) mOrange], resulting in the generation of intramolecular BRET. These LumiFluors were subsequently used for bioluminescent imaging *in vivo* where they exhibited improved luminescence and stability over Nluc alone and were successfully used for deep tissue analysis. Whereas, Chu et al. (2016) described a fusion protein called Antares (a bright orange-red fluorescent protein CyOFP1 fused to Nluc) that functioned as a sensitive reporter *in vivo* with brighter signals from deep tissues than other bioluminescent proteins.

While Schaub et al. (2015) characterized the novel LumiFluors in the context of monitoring tumorigenesis, they suggested the reporters could be used for further *in vivo* studies to monitor biological events such as PPIs and protein-ligand interactions. Following on from this study, Alcobia et al. (2018) successfully monitored protein-ligand interactions *in vivo*, demonstrating specific binding of a fluorescent propranolol ligand to Nluc-tagged β_2_AR within a mouse model of breast cancer. NanoBRET ligand binding signals were successfully monitored *in vivo* following intratumoral injection of propranolol-BY630. Importantly this response could be prevented by intratumoral or intravenous pre-treatment with the β_2_AR- selective antagonist ICI 118551. This study demonstrates the exciting potential of NanoBRET for use in *in vivo* settings.

A limitation that Nluc may face in *in vivo* studies is its blue wavelength spectrum, as red-shifted spectra are optimal for use *in vivo* due to tissue absorbing and scattering blue light (Stacer et al., 2013; Schaub et al., 2015). Indeed, Schaub et al. (2015) comment that their red-shifted (mOrange) LumiFluor exhibited greater signal *in vivo* than the GFP-based LumiFluor, despite exhibiting equivalent signals *in vitro*, and attribute this to the red wavelength emissions allowing for greater tissue penetrance. In addition to the use of the intramolecular LumiFluors, this limitation may be overcome by the development of furimazine analogs that show significant red-shift of Nluc's emission spectrum as reported by Shakhmin et al. (2017). Indeed, Yeh et al. (2017) developed a mutant of Nluc (Nluc-D19S/D85N/C164H) termed teLuc for its teal peak emission (502 nm) when coupled with the coelenterazine analog, diphenylterazine. TeLuc coupled with diphenylterazine was also shown to be brighter than Nluc with furimazine both *in vitro* and *in vivo*, though not as bright as the Antares luciferase-fluorescent protein fusion described by Chu et al. (2016).

The extension of NanoBRET, including NanoBRET-based ligand binding studies, into *in vivo* settings is an exciting technological advance with vast implications to the field of receptor pharmacology.

## NanoLuc Binary Technology (NanoBiT)

Protein-fragment complementation assays (PCAs) are assays in which a reporter, such as a luciferase or fluorophore, is split into complementary fragments and fused to proteins of interest, enabling monitoring of PPIs (Michnick et al., 2007). The fragments will only exhibit significant signal when brought into close proximity through interaction of the complementary fused proteins. Although this method has been used extensively (Ozawa, 2006; Michnick et al., 2007; Massoud et al., 2010), confounding experimental issues arise from the possibility of alterations to PPIs due to the intrinsic affinity between complementary reporter fragments, as well as steric hindrance as a result of fragment size (Dixon et al., 2015). Nluc has been investigated by Dixon et al. (2015) for its potential as a PCA that could overcome these limitations with appropriate optimization of the complementary fragments. This has led to the development of the Nluc-derived split luciferase reporter system named NanoBiT. NanoBiT comprises an 18 kDa Nluc fragment (LgBiT) and a 1.3 kDa fragment (SmBiT). LgBiT has been optimized to exhibit high stability leading to improved expression levels and performance at physiological levels, while SmBiT has been optimized to produce low intrinsic affinity (*K*_D_ = 190 μM) with the complementary fragment (Dixon et al., 2015). This enables NanoBiT to be used to investigate weak PPIs (of binding affinity values up to ~10 μM) (Dixon et al., 2015), as the PPI will not be overwhelmed by the intrinsic affinity of the complementary fragments. Additionally, a fragment termed HiBiT that exhibits high affinity (*K*_D =_ 700 pM) for LgBiT has also been described, which broadens the potential of NanoBiT technology to further novel applications (Schwinn et al., 2018).

Early applications of NanoBiT demonstrated its utility to detect PPIs. Oh-hashi et al. (2016) used NanoBiT to investigate PPIs of two proteins implicated in familial amyotrophic lateral sclerosis (ALS). Using fusion constructs tagged with SmBiT and LgBiT, the homodimerization of superoxide dismutase 1 (SOD1) proteins was successfully observed, along with disruptions to homodimerization through mutation of the SOD1 proteins. Homodimerization of the TDP43 protein was also investigated using NanoBiT, as were interactions between SOD1 and TDP43. Observations of SOD1 homodimerization and the subsequent effects of SOD1 mutations on dimerization were consistent with previous observations using bimolecular fluorescence complementation, fluorescence lifetime imaging, and fluorescence correlation spectroscopy (Kim et al., 2014). However, when using NanoBiT to investigate aggregation of mutated SOD1, no signal from the reconstituted luciferase was observed. This differs from previous findings that indicate mutated SOD1 proteins aggregate (Matsumoto et al., 2005), which would suggest that reconstitution of the fragmented luciferase should be observable. Oh-hashi et al. (2016) suggests that this discrepancy may be attributable to the structure of the mutated SOD1 aggregates leading to unfavorable orientations of the NanoBiT fragments that are not compatible with luciferase reconstitution and therefore subsequent luciferase activity. While acknowledging that this demonstrates the high level of sensitivity achieved when using NanoBiT, Oh-hashi et al. (2016) also acknowledge that this high specificity can be a limitation under certain conditions, and this needs to be considered when deciding if NanoBiT is an appropriate technique to use in one's own experiments.

NanoBiT has also been used to investigate GPCR pharmacology. In the original description of NanoBiT, Dixon et al. (2015) monitored β-arrestin2-LgBiT or β-arrestin2-SmBiT recruitment to vasopressin V_2_ receptor-SmBiT or β_2_AR-LgBiT by Nluc complementation, respectively. Additionally, Storme et al. (2018) used NanoBiT to investigate β-arrestin2 interactions with the adenosine A_3_ receptor. Fusion constructs of mutated A_3_ receptors with SmBiT or LgBiT tags were produced and included a C′-terminal tail truncation as well as phosphorylation site mutants and mutants with alterations to the highly conserved DRY motif that plays a critical role in receptor activation. Using an optimized system of the A_3_ receptor C′-terminally-tagged with LgBiT and β-arrestin2 N′-terminally-tagged with SmBiT, Storme et al. (2018) were able to successfully observe β-arrestin2 recruitment to an array of A_3_ mutant receptors. They found, unexpectedly, that phosphorylation of intracellular sites was not critical for β-arrestin2 recruitment to the A_3_ receptor. The same group has previously published the use of NanoBiT to study β-arrestin2 recruitment to cannabinoid receptors following stimulation by synthetic cannabinoids and their metabolites (Cannaert et al., 2016). Their results suggest that some synthetic cannabinoid metabolites act as agonists at cannabinoid receptors. NanoBiT has also recently been used to investigate β-arrestin2 recruitment to classical and atypical chemokine receptors following stimulation by an array of chemokine-derived peptides (Szpakowska et al., 2018).

Additional recent studies by Dupuis et al. (2017) and Reyes-Alcaraz et al. (2018) demonstrate the utility of the NanoBiT system for monitoring GPCR-β-arrestin interactions that are important for GPCR pharmacology. NanoBiT complementation assays have also been configured to investigate other GPCR signaling processes. Bodle et al. (2017) used NanoBiT complementation to investigate interactions between regulator of G protein signaling (RGS) proteins and G protein α or β subunits. Importantly, they found luminescence generated by RGS4-Gα_i1_ complementation could be prevented by a known RGS4 inhibitor and that the assay could be configured as an RGS screen with a maximal Z′ factor of 0.73 (Z′ factor = 1 – [(3 SD of positive control + 3 SD of negative control)/(mean of positive control – mean of negative control)]) (Zhang et al., 1999). Whereas, Wan et al. (2018) investigated β_2_AR and endothelin-A receptor mediated G protein recruitment using mini G_s_, G_si_, G_sq_, or G_12_ proteins and NanoBiT complementation. In their study they found the most efficient complementation took place when LgBiT was fused to the mini G proteins and SmBiT was fused to the receptor, and recruitment was ligand and concentration dependent. Expanding on these studies, Laschet et al. (2019), developed a NanoBiT complementation assay to monitor GPCR interactions with full length G proteins (Gα_i1_, Gα_i2_, Gα_i3_, Gα_o_, Gα_s_, Gα_q_ Gα_11_, Gα_12_, and Gα_13_) tagged with LgBiT. The authors initially tested the Gα_i_-coupled dopamine D_2_ receptor, histamine H_3_ receptor and the Succinate receptor but observed only low level signal for receptors tagged on the C-terminus with the SmBiT peptide. However, when substituted with the 13 amino acid native peptide fragment (K_D_ = 0.9 μM) a marked increase in signal amplitude and stability was observed, which importantly was reversible, indicating the affinity of the native peptide-LgBiT complementation did not affect the PPI. This system of complementation between native peptide-tagged receptors and LgBiT-tagged G proteins was also used to investigate interactions at the β_2_AR, histamine H_1_ receptor and thromboxane A_2_ receptor. An important consideration of this system is placement of the LgBiT fragment within the G protein, as the authors demonstrated that suboptimal placement leads to poor or complete loss of luciferase complementation.

While the NanoBiT system has most frequently been used as a tool to directly monitor PPIs through PCA (Dixon et al., 2015; Hu et al., 2018), it has also been used in conjunction with a fluorophore to produce BRET. Schwinn et al. (2018) monitored BRET between the reconstituted luciferase subunits (LgBiT and HiBiT) and a fluorophore-antibody conjugate to observe post-translational hydroxylation of hypoxia-inducible factor 1A (HIF1α). They were able to simultaneously measure the amount of hydroxylated HIF1α through the BRET ratio as well as the total amount of HIF1α present by measuring the luminescence output from the HiBiT tagged HIF1α proteins. This study demonstrates the versatility of NanoBiT and its potentially broad applicability in a variety of biophysical assays.

## CRISPR/Cas9 Genome Engineering and BRET

**Figure 3:**
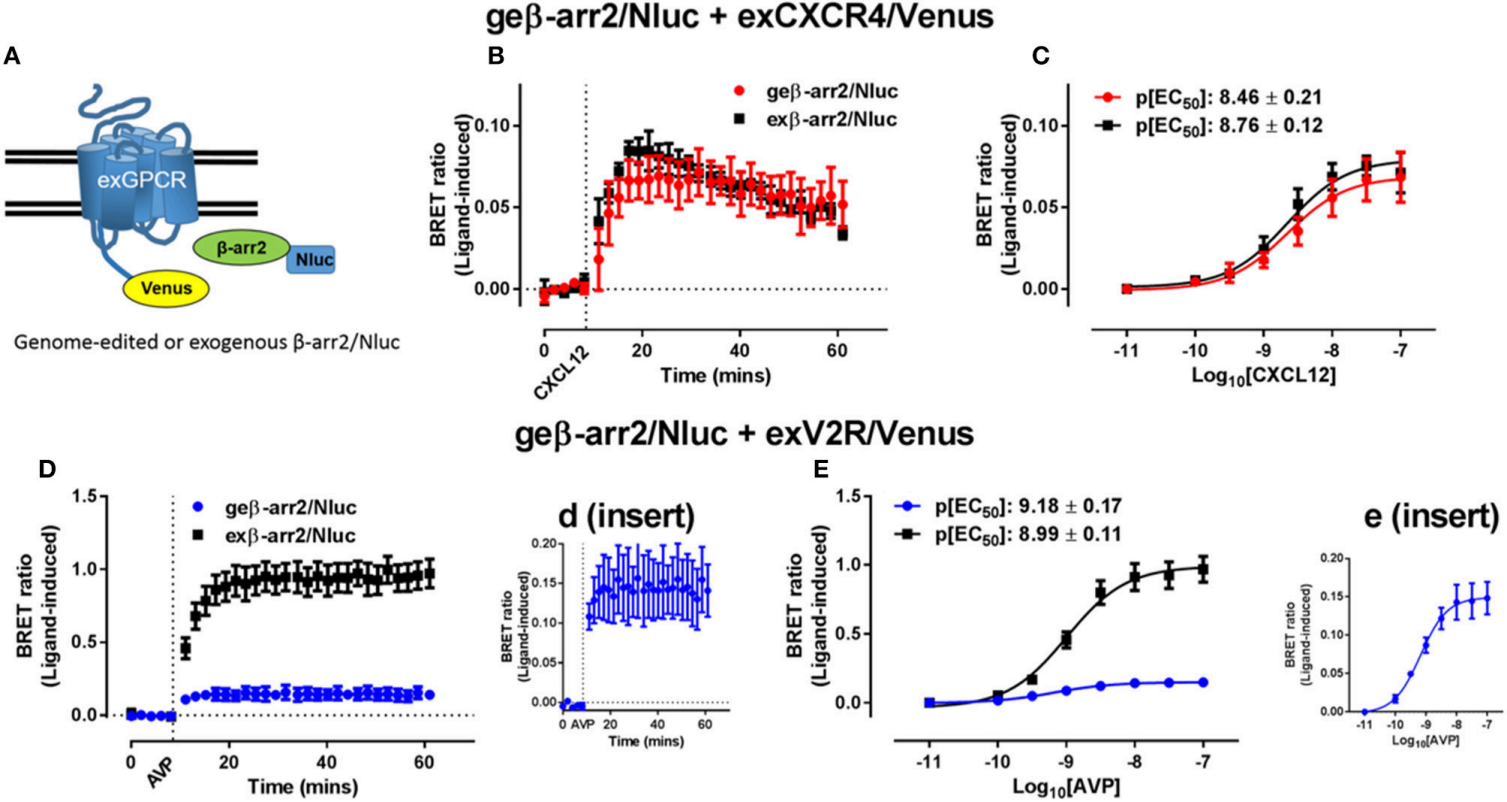
Investigating recruitment of genome-edited β-arrestin2 using BRET. **(A)** Schematic representation of the exogenously expressed GPCR fused to Venus (exGPCR/Venus) and β-arr2/Nluc BRET configuration. HEK293FT cells expressing genome-edited β-arrestin2 fused to Nluc (geβ-arr2/Nluc) transiently transfected with cDNA coding for **(B,C)** CXCR4 fused to Venus (exCXCR4/Venus; red circles) or **(D,E)** V2R fused to Venus (exV2R/Venus, blue circles) as well as HEK293FT cells transiently co-transfected to express exogenous β-arrestin2 fused to Nluc (exβ-arr2/Nluc, black squares) at near endogenous levels and **(B,C)** exCXCR4/Venus or **(D,E)** exV2R/Venus. **(B,D)** Kinetic profiles of β-arrestin2/Nluc recruitment initiated by addition of CXCL12 (30 nM) or AVP (100 nM) for CXCR4 and V2R, respectively. Concentration-dependent recruitment of genome-edited or exogenous β arrestin2/Nluc to **(C)** exCXCR4/Venus or **(E)** exV2R/Venus mediated by CXCL12 (10 pM−100 nM) or AVP (10 pM−100 nM), respectively. Inserts **(d,e)** show expanded view of geβ-arr2/Nluc recruitment to exV2R/Venus presented in **(D,E)**. Points and bars represent mean ± S.E.M. of three or four independent experiments. p[EC_50_] = –log_10_ half maximal effective concentration. Reproduced and modified from White et al. (2017) under a Creative Commons Attribution 4.0 International License. Full terms provided at http://creativecommons.org/licenses/by/4.0/.

Due to their relative ease of use and versatility, CRISPR/Cas9 systems have revolutionized genome engineering (Jinek et al., 2012; Cong et al., 2013). The first use of CRISPR to perform a BRET assay was reported by White et al. (2017), where successful observation of a BRET signal was achieved using proteins expressed under endogenous promotion tagged with Nluc through the use of CRISPR/Cas9-mediated homology-directed repair (Doudna and Charpentier, 2014). White et al. (2017) used NanoBRET to investigate recruitment of genome-edited β-arrestin2 and compared this to recruitment of exogenously-expressed (transfected) β-arrestin2 to multiple GPCRs (Figure 3). Minimal differences in signal between genome-edited and exogenously expressed β-arrestin2 were observed for some receptors such as CXCR4 that are thought to interact with β-arrestin2 somewhat transiently (Figures 3B,C), whereas for vasopressin V_2_ receptors that form more stable complexes with β-arrestin2 (Figures 3D,E) a large difference in signal magnitude between genome-edited and exogenously expressed β-arrestin2 was observed. In addition, it was possible to observe recruitment of exogenous β-arrestin2 to genome-edited CXCR4 fused to Nluc as well as trafficking and internalization of CXCR4. Highlighting the potential importance of being able to use genome-edited proteins, depending on whether CXCR4 or β-arrestin2 was overexpressed, different kinetic and pharmacological parameters were observed. While this advance in BRET technology has been aided by CRISPR/Cas9 genome engineering, Nluc is also important for the approach. As shown by White et al. (2017), when Rluc8 was introduced into the gene in place of Nluc, the Z′ factor decreased substantially, indicating an increase in assay variability. This is likely due to the increased luminescence of Nluc allowing for detection of the low protein levels found when expressed under endogenous promotion. The viability and utility of combining CRISPR/Cas9 technologies with Nluc or NanoBiT was further demonstrated by Oh-hashi et al. (2017) and Schwinn et al. (2018) who used a similar technique for endogenous tagging of proteins of interest with the HiBiT peptide to form part of the NanoBiT split luciferase reporter system. Vasta et al. (2018) also utilized CRISPR/Cas9 techniques to produce a Cyclin-Dependent Kinase 2 (CDK2)-Nluc fusion protein that were subsequently used to investigate kinase inhibitor binding at endogenous expression levels. Very recently, White et al. (2019) used CRISPR/Cas9 genome engineering to insert full length Nluc onto the N′-terminus of the adenosine A_2B_ receptor expressed in HEK293 cells. Despite finding exceptionally low levels of Nluc-tagged receptor expression, these cells could readily be used in NanoBRET ligand binding assays.

Although there are multiple studies demonstrating successful tagging of endogenous loci with luciferases or fluorescent proteins (Lackner et al., 2015; Ratz et al., 2015), to date few have used these systems to conduct BRET assays (White et al., 2017, 2019; Schwinn et al., 2018). White et al. (2017) not only successfully demonstrated the use of CRISPR/Cas9 engineered proteins in NanoBRET assays, but also showed differential pharmacology of GPCR-β-arrestin2 interactions depending on the level of protein expression. The potential advantage of using proteins expressed under endogenous promotion has recently been highlighted in studies of toll-like receptors, where constitutive PPIs detected using traditional BRET techniques and transient transfection were potentially a result of protein overexpression and not of physiologically-relevant interaction (Sampaio et al., 2018). The combination of CRISPR/Cas9 and NanoBRET will likely allow the investigation of physiology that was previously difficult to interrogate using traditional BRET techniques.

## Conclusions

BRET and NanoBRET are powerful, real-time profiling tools for monitoring PPIs and ligand-protein interactions. When used in conjunction with other innovative biological tools such as fluorescent dyes and CRISPR/Cas9 genome engineering, NanoBRET in particular has proven to be extremely versatile, leading to the development of cutting edge assays such as receptor-ligand binding and BRET with endogenously expressed proteins. Both these examples illustrate the increasingly broad applications that can be undertaken with NanoBRET assays. Moreover, the use of NanoBRET with endogenous proteins allows assays to be conducted in more physiologically-relevant conditions, further enhancing the BRET approach as a tool to interrogate cellular biology and molecular pharmacology.

## Author Contributions

All authors listed have made a substantial, direct and intellectual contribution to the work, and approved it for publication.

### Conflict of Interest Statement

KP receives funding from Promega, BMG Labtech, and Dimerix as Australian Research Council Linkage Grant participating organizations. These participating organizations played no role in writing or editing of the manuscript. KP is Chief Scientific Advisor of Dimerix, of which he maintains a shareholding. The remaining authors declare that the article was written in the absence of any commercial or financial relationships that could be construed as a potential conflict of interest.

## References

[B1] Albizu L. Cottet M. Kralikova M. Stoev S. Seyer R. Brabet I.. (2010). Time-resolved FRET between GPCR ligands reveals oligomers in native tissues. Nat. Chem. Biol. 6, 587–594. 10.1038/nchembio.396 20622858 PMC3506176

[B2] Alcobia D. C. Ziegler A. I. Kondrashov A. Comeo E. Mistry S. Kellam B.. (2018). Visualizing ligand binding to a GPCR *in vivo* using NanoBRET. iScience 6, 280–288. 10.1016/j.isci.2018.08.006 30240618 PMC6137713

[B3] Anindita P. D. Sasaki M. Nobori H. Sato A. Carr M. Ito N.. (2016). Generation of recombinant rabies viruses encoding NanoLuc luciferase for antiviral activity assays. Virus Res. 215, 121–128. 10.1016/j.virusres.2016.02.002 26869397

[B4] Azevedo M. F. Nie C. Q. Elsworth B. Charnaud S. C. Sanders P. R. Crabb B. S.. (2014). Plasmodium falciparum transfected with ultra bright NanoLuc luciferase offers high sensitivity detection for the screening of growth and cellular trafficking inhibitors. PLoS ONE 9:e112571. 10.1371/journal.pone.0112571 25392998 PMC4231029

[B5] Bodle C. R. Hayes M. P. O'Brien J. B. Roman D. L. (2017). Development of a bimolecular luminescence complementation assay for RGS: G protein interactions in cells. Anal. Biochem. 522, 10–17. 10.1016/j.ab.2017.01.013 28115169 PMC5330260

[B6] Boute N. Lowe P. Berger S. Malissard M. Robert A. Tesar M. (2016). NanoLuc luciferase–a multifunctional tool for high throughput antibody screening. Front. Pharmacol. 7:27. 10.3389/fphar.2016.00027 26924984 PMC4758271

[B7] Cannaert A. Storme J. Franz F. Auwärter V. Stove C. P. (2016). Detection and activity profiling of synthetic cannabinoids and their metabolites with a newly developed bioassay. Anal. Chem. 88, 11476–11485. 10.1021/acs.analchem.6b02600 27779402

[B8] Christiansen E. Hudson B. D. Hansen A. H. Milligan G. Ulven T. (2016). Development and characterization of a potent free fatty acid receptor 1 (FFA1) fluorescent tracer. J. Med. Chem. 59, 4849–4858. 10.1021/acs.jmedchem.6b00202 27074625

[B9] Chu J. Oh Y. Sens A. Ataie N. Dana H. Macklin J. J.. (2016). A bright cyan-excitable orange fluorescent protein facilitates dual-emission microscopy and enhances bioluminescence imaging *in vivo*. Nat. Biotechnol. 34, 760–767. 10.1038/nbt.3550 27240196 PMC4942401

[B10] Cong L. Ran F. A. Cox D. Lin S. Barretto R. Habib N.. (2013). Multiplex genome engineering using CRISPR/Cas systems. Science 339, 819–823. 10.1126/science.1231143 23287718 PMC3795411

[B11] Conroy S. Kindon N. D. Glenn J. Stoddart L. A. Lewis R. J. Hill S. J. (2018). Synthesis and evaluation of the first fluorescent antagonists of the human P2Y2 receptor based on AR-C118925. J. Med. Chem. 61, 3089–3113. 10.1021/acs.jmedchem.8b00139 29558126 PMC6026847

[B12] Contag C. H. Spilman S. D. Contag P. R. Oshiro M. Eames B. Dennery P.. (1997). Visualizing gene expression in living mammals using a bioluminescent reporter. Photochem. Photobiol. 66, 523–531. 10.1111/j.1751-1097.1997.tb03184.x 9337626

[B13] Dacres H. Michie M. Wang J. Pfleger K. D. Trowell S. C. (2012). Effect of enhanced Renilla luciferase and fluorescent protein variants on the Förster distance of Bioluminescence resonance energy transfer (BRET). Biochem. Biophys. Res. Commun. 425, 625–629. 10.1016/j.bbrc.2012.07.133 22877756

[B14] De Niz M. Stanway R. R. Wacker R. Keller D. Heussler V. T. (2016). An ultrasensitive NanoLuc-based luminescence system for monitoring *Plasmodium berghei* throughout its life cycle. Malaria J. 15:232. 10.1186/s12936-016-1291-9 27102897 PMC4840902

[B15] den Hamer A. Dierickx P. Arts R. de Vries J. S. M. Brunsveld L. Merkx M. (2017). Bright bioluminescent BRET sensor proteins for measuring intracellular caspase activity. ACS Sens. 2, 729–734. 10.1021/acssensors.7b00239 28670623 PMC5485374

[B16] Dixon A. S. Schwinn M. K. Hall M. P. Zimmerman K. Otto P. Lubben T. H.. (2015). NanoLuc complementation reporter optimized for accurate measurement of protein interactions in cells. ACS Chem. Biol. 11, 400–408. 10.1021/acschembio.5b00753 26569370

[B17] Doudna J. A. Charpentier E. (2014). The new frontier of genome engineering with CRISPR-Cas9. Science 346:1258096. 10.1126/science.1258096 25430774

[B18] Dupuis N. Laschet C. Franssen D. Szpakowska M. Gilissen J. Geubelle P.. (2017). Activation of the orphan G protein-coupled receptor GPR27 by surrogate ligands promotes β-arrestin 2 recruitment. Mol. Pharmacol. 91, 595–608. 10.1124/mol.116.107714 28314853

[B19] England C. G. Ehlerding E. B. Cai W. (2016). NanoLuc: a small luciferase is brightening up the field of bioluminescence. Bioconjug. Chem. 27, 1175–1187. 10.1021/acs.bioconjchem.6b00112 27045664 PMC4871753

[B20] Eyre N. S. Aloia A. L. Joyce M. A. Chulanetra M. Tyrrell D. L. Beard M. R. (2017). Sensitive luminescent reporter viruses reveal appreciable release of hepatitis C virus NS5A protein into the extracellular environment. Virology 507, 20–31. 10.1016/j.virol.2017.04.003 28395182

[B21] Fraga H. (2008). Firefly luminescence: a historical perspective and recent developments. Photochem. Photobiol. Sci. 7, 146–158. 10.1039/b719181b 18264582

[B22] Gonçalves M. S. T. (2008). Fluorescent labeling of biomolecules with organic probes. Chem. Rev. 109, 190–212. 10.1021/cr0783840 19105748

[B23] Goyet E. Bouquier N. Ollendorff V. Perroy J. (2016). Fast and high resolution single-cell BRET imaging. Sci. Rep. 6:28231. 10.1038/srep28231 27302735 PMC4908377

[B24] Hall M. P. Unch J. Binkowski B. F. Valley M. P. Butler B. L. Wood M. G.. (2012). Engineered luciferase reporter from a deep sea shrimp utilizing a novel imidazopyrazinone substrate. ACS Chem. Biol. 7, 1848–1857. 10.1021/cb3002478 22894855 PMC3501149

[B25] Hamdan F. F. Audet M. Garneau P. Pelletier J. Bouvier M. (2005). High-throughput screening of G protein-coupled receptor antagonists using a bioluminescence resonance energy transfer 1-based β-arrestin2 recruitment assay. J. Biomol. Screen. 10, 463–475. 10.1177/1087057105275344 16093556

[B26] Hamdan F. F. Percherancier Y. Breton B. Bouvier M. (2006). Monitoring protein-protein interactions in living cells by bioluminescence resonance energy transfer (BRET). Curr. Protoc. Neurosci. 34, 5–23. 10.1002/0471142301.ns0523s34 18428639

[B27] Hansen A. H. Sergeev E. Pandey S. K. Hudson B. D. Christiansen E. Milligan G.. (2017). Development and characterization of a fluorescent tracer for the free fatty acid receptor 2 (FFA2/GPR43). J. Med. Chem. 60, 5638–5645. 10.1021/acs.jmedchem.7b00338 28570808

[B28] Hattori M. Kawamura G. Kojima R. Kamiya M. Urano Y. Ozawa T. (2016). Confocal bioluminescence imaging for living tissues with a caged substrate of luciferin. Anal. Chem. 88, 6231–6238. 10.1021/acs.analchem.5b04142 27216493

[B29] Heise K. Oppermann H. Meixensberger J. Gebhardt R. Gaunitz F. (2013). Dual luciferase assay for secreted luciferases based on Gaussia and NanoLuc. Assay Drug Dev. Technol. 11, 244–252. 10.1089/adt.2013.509 23679848

[B30] Hiblot J. Yu Q. Sabbadini M. D. Reymond L. Xue L. Schena A. (2017). Luciferases with tunable emission wavelengths. Angew. Chem. Int. Ed. 129, 14748–14752. 10.1002/ange.201708277 28941028

[B31] Hikiji T. Norisada J. Hirata Y. Okuda K. Nagasawa H. Ishigaki S.. (2015). A highly sensitive assay of IRE1 activity using the small luciferase NanoLuc: evaluation of ALS-related genetic and pathological factors. Biochem. Biophys. Res. Commun. 463, 881–887. 10.1016/j.bbrc.2015.05.132 26056941

[B32] Hoare B. L. Bruell S. Sethi A. Gooley P. R. Lew M. J. Hossain M. A.. (2019). Multi-component mechanism of H2 relaxin binding to RXFP1 through NanoBRET kinetic analysis. iScience 11, 93–113. 10.1016/j.isci.2018.12.004 30594862 PMC6309025

[B33] Hounsou C. Baehr C. Gasparik V. Alili D. Belhocine A. Rodriguez T.. (2017). From the promiscuous asenapine to potent fluorescent ligands acting at a series of aminergic G-Protein-Coupled Receptors. J. Med. Chem. 61, 174–188. 10.1021/acs.jmedchem.7b01220 29219316

[B34] Hu M.-J. Shao X.-X. Li H.-Z. Nie W.-H. Wang J.-H. Liu Y.-L.. (2018). Development of a novel ligand binding assay for relaxin family peptide receptor 3 and 4 using NanoLuc complementation. Amino Acids 50, 1111–1119. 10.1007/s00726-018-2588-5 29770870

[B35] Inouye S. Watanabe K. Nakamura H. Shimomura O. (2000). Secretional luciferase of the luminous shrimp *Oplophorus gracilirostris*: cDNA cloning of a novel imidazopyrazinone luciferase. FEBS Lett. 481, 19–25. 10.1016/S0014-5793(00)01963-3 10984608

[B36] Jaeger W. C. Armstrong S. P. Hill S. J. Pfleger K. D. G. (2014). Biophysical detection of diversity and bias in GPCR function. Front. Endocrinol. 5:26. 10.3389/fendo.2014.00026 24634666 PMC3943086

[B37] Jinek M. Chylinski K. Fonfara I. Hauer M. Doudna J. A. Charpentier E. (2012). A programmable dual-RNA-guided DNA endonuclease in adaptive bacterial immunity. Science 337, 816–821. 10.1126/science.1225829 22745249 PMC6286148

[B38] Karlsson E. A. Meliopoulos V. A. Savage C. Livingston B. Mehle A. Schultz-Cherry S. (2015). Visualizing real-time influenza virus infection, transmission and protection in ferrets. Nat. Commun. 6:6378. 10.1038/ncomms7378 25744559 PMC4366512

[B39] Kilpatrick L. E. Friedman-Ohana R. Alcobia D. C. Riching K. Peach C. J. Wheal A. J.. (2017). Real-time analysis of the binding of fluorescent VEGF165a to VEGFR2 in living cells: effect of receptor tyrosine kinase inhibitors and fate of internalized agonist-receptor complexes. Biochem. Pharmacol. 136, 62–75. 10.1016/j.bcp.2017.04.006 28392095 PMC5457915

[B40] Kim J. Grailhe R. (2016). Nanoluciferase signal brightness using furimazine substrates opens bioluminescence resonance energy transfer to widefield microscopy. Cytometry A 89, 742–746. 10.1002/cyto.a.22870 27144967

[B41] Kim J. Lee H. Lee J. H. Kwon D. Y. Genovesio A. Fenistein D.. (2014). Dimerization, oligomerization, and aggregation of human amyotrophic lateral sclerosis copper/zinc superoxide dismutase 1 protein mutant forms in live cells. J. Biol. Chem. 289, 15094–15103. 10.1074/jbc.M113.542613 24692554 PMC4031559

[B42] Kocan M. See H. B. Seeber R. M. Eidne K. A. Pfleger K. D. G. (2008). Demonstration of improvements to the bioluminescence resonance energy transfer (BRET) technology for the monitoring of G protein-coupled receptors in live cells. J. Biomol. Screen. 13, 888–898. 10.1177/1087057108324032 18812574

[B43] Lackner D. H. Carré A. Guzzardo P. M. Banning C. Mangena R. Henley T.. (2015). A generic strategy for CRISPR-Cas9-mediated gene tagging. Nat. Commun. 6:10237. 10.1038/ncomms10237 26674669 PMC4703899

[B44] Lan T. H. Liu Q. Li C. Wu G. Lambert N. A. (2012). Sensitive and high resolution localization and tracking of membrane proteins in live cells with BRET. Traffic 13, 1450–1456. 10.1111/j.1600-0854.2012.01401.x 22816793 PMC3889717

[B45] Laschet C. Dupuis N. Hanson J. (2019). A dynamic and screening-compatible nanoluciferase-based complementation assay enables profiling of individual GPCR-G protein interactions. J. Biol. Chem. 10.1074/jbc.RA118.006231 30593506 PMC6422084

[B46] Lichtman J. W. Conchello J.-A. (2005). Fluorescence microscopy. Nat. Methods 2:910. 10.1038/nmeth817 16299476

[B47] Liu J. O'Kane D. J. Escher A. (1997). Secretion of functional *Renilla reniformis* luciferase by mammalian cells. Gene 203, 141–148. 10.1016/S0378-1119(97)00505-2 9426244

[B48] Loh J. M. Proft T. (2014). Comparison of firefly luciferase and NanoLuc luciferase for biophotonic labeling of group A Streptococcus. Biotechnol. Lett. 36, 829–834. 10.1007/s10529-013-1423-z 24322775

[B49] Lohse M. J. Nuber S. Hoffmann C. (2012). Fluorescence/bioluminescence resonance energy transfer techniques to study G-protein-coupled receptor activation and signaling. Pharmacol. Rev. 64, 299–336. 10.1124/pr.110.004309 22407612

[B50] Lorenz W. Cormier M. O'Kane D. Hua D. Escher A. Szalay A. (1996). Expression of the *Renilla reniformis* luciferase gene in mammalian cells. J. Biolumin. Chemilumin. 11, 31–37. 10.1002/(SICI)1099-1271(199601)11:1<31::AID-BIO398>3.0.CO;2-M 8686494

[B51] Los G. V. Encell L. P. McDougall M. G. Hartzell D. D. Karassina N. Zimprich C.. (2008). HaloTag: a novel protein labeling technology for cell imaging and protein analysis. ACS Chem. Biol. 3, 373–382. 10.1021/cb800025k 18533659

[B52] Machleidt T. Woodroofe C. C. Schwinn M. K. Méndez J. Robers M. B. Zimmerman K.. (2015). NanoBRET - a novel BRET platform for the analysis of protein–protein interactions. ACS Chem. Biol. 10, 1797–1804. 10.1021/acschembio.5b00143 26006698

[B53] Masser A. E. Kandasamy G. Kaimal J. M. Andréasson C. (2016). Luciferase NanoLuc as a reporter for gene expression and protein levels in *Saccharomyces cerevisiae*. Yeast 33, 191–200. 10.1002/yea.3155 26860732 PMC5069653

[B54] Massoud T. F. Paulmurugan R. Gambhir S. S. (2010). A molecularly engineered split reporter for imaging protein-protein interactions with positron emission tomography. Nat. Med. 16:921. 10.1038/nm.2185 20639890 PMC2917476

[B55] Matsumoto G. Stojanovic A. Holmberg C. I. Kim S. Morimoto R. I. (2005). Structural properties and neuronal toxicity of amyotrophic lateral sclerosis–associated Cu/Zn superoxide dismutase 1 aggregates. J. Cell Biol. 171, 75–85. 10.1083/jcb.200504050 16216923 PMC2171239

[B56] Michnick S. W. Ear P. H. Manderson E. N. Remy I. Stefan E. (2007). Universal strategies in research and drug discovery based on protein-fragment complementation assays. Nat. Rev. Drug Discov. 6:569. 10.1038/nrd2311 17599086

[B57] Milligan G. (2004). Applications of bioluminescence-and fluorescence resonance energy transfer to drug discovery at G protein-coupled receptors. Eur. J. Pharm. Sci. 21, 397–405. 10.1016/j.ejps.2003.11.010 14998570

[B58] Mo X.-L. Luo Y. Ivanov A. A. Su R. Havel J. J. Li Z.. (2016). Enabling systematic interrogation of protein–protein interactions in live cells with a versatile ultra-high-throughput biosensor platform. J. Mol. Cell Biol. 8, 271–281. 10.1093/jmcb/mjv064 26578655 PMC4937889

[B59] Mocking T. A. Verweij E. W. Vischer H. F. Leurs R. (2018). Homogeneous, Real-time NanoBRET binding assays for the histamine H3 and H4 receptors on living cells. Mol. Pharmacol. 94, 1371–1381. 10.1124/mol.118.113373 30249614

[B60] Moreno E. Canet J. Gracia E. Lluís C. Mallol J. Canela E. I.. (2018). Molecular evidence of adenosine deaminase linking adenosine A2A receptor and CD26 proteins. Front. Pharmacol. 9:106. 10.3389/fphar.2018.00106 29497379 PMC5818423

[B61] Morishima S. Suzuki F. Nishimune A. Yoshiki H. Akino H. Yokoyama O.. (2010). Visualization and tissue distribution of α1L-adrenoceptor in human prostate by the fluorescently labeled ligand Alexa-488-silodosin. J. Urol. 183, 812–819. 10.1016/j.juro.2009.09.078 20034639

[B62] Moriya C. Taniguchi H. Nagatoishi S. Igarashi H. Tsumoto K. Imai K. (2018). PRDM14 directly interacts with heat shock proteins HSP90α and glucose-regulated protein 78. Cancer Sci. 109, 373–383. 10.1111/cas.13458 29178343 PMC5797828

[B63] Oh-hashi K. Furuta E. Fujimura K. Hirata Y. (2017). Application of a novel HiBiT peptide tag for monitoring ATF4 protein expression in Neuro2a cells. Biochem. Biophys. Rep. 12, 40–45. 10.1016/j.bbrep.2017.08.002 28955790 PMC5613219

[B64] Oh-hashi K. Hirata Y. Kiuchi K. (2016). SOD1 dimerization monitoring using a novel split NanoLuc, NanoBit. Cell Biochem. Funct. 34, 497–504. 10.1002/cbf.3222 27687581

[B65] Ozawa T. (2006). Designing split reporter proteins for analytical tools. Anal. Chim. Acta 556, 58–68. 10.1016/j.aca.2005.06.026 17723331

[B66] Peach C. J. Kilpatrick L. E. Friedman-Ohana R. Zimmerman K. Robers M. B. Wood K. V.. (2018). Real-time ligand binding of fluorescent VEGF-A isoforms that discriminate between VEGFR2 and NRP1 in living cells. Cell Chem. Biol. 25, 1208–1218. 10.1016/j.chembiol.2018.06.012 30057299 PMC6200776

[B67] Pfleger K. D. Dromey J. R. Dalrymple M. B. Lim E. M. Thomas W. G. Eidne K. A. (2006a). Extended bioluminescence resonance energy transfer (eBRET) for monitoring prolonged protein–protein interactions in live cells. Cell. Signal. 18, 1664–1670. 10.1016/j.cellsig.2006.01.004 16492395

[B68] Pfleger K. D. Eidne K. A. (2005). Monitoring the formation of dynamic G-protein-coupled receptor–protein complexes in living cells. Biochem. J. 385, 625–637. 10.1042/BJ20041361 15504107 PMC1134737

[B69] Pfleger K. D. Eidne K. A. (2006). Illuminating insights into protein-protein interactions using bioluminescence resonance energy transfer (BRET). Nat. Methods 3:165. 10.1038/nmeth841 16489332

[B70] Pfleger K. D. Seeber R. M. Eidne K. A. (2006b). Bioluminescence resonance energy transfer (BRET) for the real-time detection of protein-protein interactions. Nat. Protoc. 1:337. 10.1038/nprot.2006.52 17406254

[B71] Prinz A. Diskar M. Herberg F. W. (2006). Application of bioluminescence resonance energy transfer (BRET) for biomolecular interaction studies. ChemBioChem 7, 1007–1012. 10.1002/cbic.200600048 16755626

[B72] Ratz M. Testa I. Hell S. W. Jakobs S. (2015). CRISPR/Cas9-mediated endogenous protein tagging for RESOLFT super-resolution microscopy of living human cells. Sci. Rep. 5:9592. 10.1038/srep09592 25892259 PMC4402611

[B73] Reyes-Alcaraz A. Lee Y. N. Yun S. Hwang J. I. Seong J. Y. (2018). Conformational signatures in β-arrestin2 reveal natural biased agonism at a G-protein-coupled receptor. Commun. Biol. 1:128. 10.1038/s42003-018-0134-3 30272007 PMC6123711

[B74] Robers M. B. Dart M. L. Woodroofe C. C. Zimprich C. A. Kirkland T. A. Machleidt T.. (2015). Target engagement and drug residence time can be observed in living cells with BRET. Nat. Commun. 6:10091. 10.1038/ncomms10091 26631872 PMC4686764

[B75] Rumyantsev K. A. Turoverov K. K. Verkhusha V. V. (2016). Near-infrared bioluminescent proteins for two-color multimodal imaging. Sci. Rep. 6:36588. 10.1038/srep36588 27833162 PMC5105121

[B76] Saito K. Chang Y. F. Horikawa K. Hatsugai N. Higuchi Y. Hashida M.. (2012). Luminescent proteins for high-speed single-cell and whole-body imaging. Nat. Commun. 3:1262. 10.1038/ncomms2248 23232392 PMC3535334

[B77] Sampaio N. G. Kocan M. Schofield L. Pfleger K. D. Eriksson E. M. (2018). Investigation of interactions between TLR2, MyD88 and TIRAP by bioluminescence resonance energy transfer is hampered by artefacts of protein overexpression. PLoS ONE 13:e0202408. 10.1371/journal.pone.0202408 30138457 PMC6107161

[B78] Schaub F. X. Reza M. S. Flaveny C. A. Li W. Musicant A. M. Hoxha S.. (2015). Fluorophore-NanoLuc BRET reporters enable sensitive *in vivo* optical imaging and flow cytometry for monitoring tumorigenesis. Cancer Res. 75, 5023–5033. 10.1158/0008-5472.CAN-14-3538 26424696 PMC4668208

[B79] Schwinn M. K. Machleidt T. Zimmerman K. Eggers C. T. Dixon A. S. Hurst R.. (2018). CRISPR-mediated tagging of endogenous proteins with a luminescent peptide. ACS Chem. Biol. 13, 467–474. 10.1021/acschembio.7b00549 28892606

[B80] Shakhmin A. Hall M. P. Machleidt T. Walker J. R. Wood K. V. Kirkland T. A. (2017). Coelenterazine analogues emit red-shifted bioluminescence with NanoLuc. Org. Biomol. Chem. 15, 8559–8567. 10.1039/C7OB01985H 28972606

[B81] Shigeto H. Ikeda T. Kuroda A. Funabashi H. (2015). A BRET-based homogeneous insulin assay using interacting domains in the primary binding site of the insulin receptor. Anal. Chem. 87, 2764–2770. 10.1021/ac504063x 25655236

[B82] Soave M. Stoddart L. A. Brown A. Woolard J. Hill S. J. (2016). Use of a new proximity assay (NanoBRET) to investigate the ligand-binding characteristics of three fluorescent ligands to the human β1-adrenoceptor expressed in HEK-293 cells. Pharmacol. Res. Perspect. 4:e00250. 10.1002/prp2.250 27588207 PMC4988514

[B83] Stacer A. C. Nyati S. Moudgil P. Iyengar R. Luker K. E. Rehemtulla A.. (2013). NanoLuc reporter for dual luciferase imaging in living animals. Mol. Imaging 12, 457–469. 10.2310/7290.2013.00062 24371848 PMC4144862

[B84] Stoddart L. A. Johnstone E. K. Wheal A. J. Goulding J. Robers M. B. Machleidt T.. (2015). Application of BRET to monitor ligand binding to GPCRs. Nat. Methods 12, 661–663. 10.1038/nmeth.3398 26030448 PMC4488387

[B85] Stoddart L. A. Kilpatrick L. E. Hill S. J. (2017). NanoBRET approaches to study ligand binding to GPCRs and RTKs. Trends Pharmacol. Sci. 39, 136–147. 10.1016/j.tips.2017.10.006 29132917

[B86] Stoddart L. A. Vernall A. J. Bouzo-Lorenzo M. Bosma R. Kooistra A. J. De Graaf C. (2018). Development of novel fluorescent histamine H 1-receptor antagonists to study ligand-binding kinetics in living cells. Sci. Rep. 8:1572 10.1038/s41598-018-19714-2 29371669 PMC5785503

[B87] Stoddart L. A. White C. W. Nguyen K. Hill S. J. Pfleger K. D. G. (2016). Fluorescence- and bioluminescence-based approaches to study GPCR ligand binding. Br. J. Pharmacol. 173, 3028–3037. 10.1111/bph.13316 26317175 PMC5125978

[B88] Storme J. Cannaert A. Van Craenenbroeck K. Stove C. P. (2018). Molecular dissection of the human A3 adenosine receptor coupling with β-arrestin2. Biochem. Pharmacol. 148, 298–307. 10.1016/j.bcp.2018.01.008 29309765

[B89] Szpakowska M. Nevins A. M. Meyrath M. Rhainds D. D'huys T. Guité-Vinet F.. (2018). Different contributions of chemokine N-terminal features attest to a different ligand binding mode and a bias towards activation of ACKR3/CXCR7 compared with CXCR4 and CXCR3. Br. J. Pharmacol. 175, 1419–1438. 10.1111/bph.14132 29272550 PMC5900987

[B90] Tiulpakov A. White C. W. Abhayawardana R. S. See H. B. Chan A. S. Seeber R. M.. (2016). Mutations of vasopressin receptor 2 including novel L312S have differential effects on trafficking. Mol. Endocrinol. 30, 889–904. 10.1210/me.2016-1002 27355191 PMC4965841

[B91] Vasta J. D. Corona C. R. Wilkinson J. Zimprich C. A. Hartnett J. R. Ingold M. R.. (2018). Quantitative, wide-spectrum kinase profiling in live cells for assessing the effect of cellular ATP on target engagement. Cell Chem. Biol. 25, 206–214. 10.1016/j.chembiol.2017.10.010 29174542 PMC5814754

[B92] Vernall A. J. Hill S. J. Kellam B. (2014). The evolving small-molecule fluorescent-conjugate toolbox for Class A GPCRs. Br. J. Pharmacol. 171, 1073–1084. 10.1111/bph.12265 23734587 PMC3952789

[B93] Vernall A. J. Stoddart L. A. Briddon S. J. Hill S. J. Kellam B. (2012). Highly potent and selective fluorescent antagonists of the human adenosine A3 receptor based on the 1, 2, 4-triazolo [4, 3-a] quinoxalin-1-one scaffold. J. Med. Chem. 55, 1771–1782. 10.1021/jm201722y 22277057

[B94] Vernall A. J. Stoddart L. A. Briddon S. J. Ng H. W. Laughton C. A. Doughty S. W. (2013). Conversion of a non-selective adenosine receptor antagonist into A 3-selective high affinity fluorescent probes using peptide-based linkers. Org. Biomol. Chem. 11, 5673–5682. 10.1039/c3ob41221k 23881285

[B95] Wan Q. Okashah N. Inoue A. Nehmé R. Carpenter B. Tate C. G.. (2018). Mini G protein probes for active G protein-coupled receptors (GPCRs) in live cells. J. Biol. Chem. 293, 7466–7473. 10.1074/jbc.RA118.001975 29523687 PMC5949987

[B96] Wang J.-H. Shao X.-X. Hu M.-J. Wei D. Liu Y.-L. Xu Z.-G.. (2017). A novel BRET-based binding assay for interaction studies of relaxin family peptide receptor 3 with its ligands. Amino Acids 49, 895–903. 10.1007/s00726-017-2387-4 28161795

[B97] White C. W. Johnstone E. K. M. See H. B. Pfleger K. D. G. (2019). NanoBRET ligand binding at a GPCR under endogenous promotion facilitated by CRISPR/Cas9 genome editing. Cell. Signal. 54, 27–34. 10.1016/j.cellsig.2018.11.018 30471466

[B98] White C. W. Vanyai H. K. See H. B. Johnstone E. K. M. Pfleger K. D. G. (2017). Using nanoBRET and CRISPR/Cas9 to monitor proximity to a genome-edited protein in real-time. Sci. Rep. 7:3187. 10.1038/s41598-017-03486-2 28600500 PMC5466623

[B99] Wood K. V. Lam Y. A. McElroy W. D. (1989). Introduction to beetle luciferases and their applications. J. Biolumin. Chemilumin. 4, 289–301. 10.1002/bio.1170040141 2678917

[B100] Wu B. Wang J. Zhao Y. Guo W. (2015). Biochemical analysis of Rabin8, the guanine nucleotide exchange factor for Rab8. Methods Cell Biol. 130, 59–68. 10.1016/bs.mcb.2015.06.018 26360028

[B101] Wu P. Brand L. (1994). Resonance energy transfer: methods and applications. Anal. Biochem. 218, 1–13. 10.1006/abio.1994.1134 8053542

[B102] Xu Y. Piston D. W. Johnson C. H. (1999). A bioluminescence resonance energy transfer (BRET) system: application to interacting circadian clock proteins. Proc. Natl. Acad. Sci. U.S.A. 96, 151–156. 10.1073/pnas.96.1.151 9874787 PMC15108

[B103] Yang J. Cumberbatch D. Centanni S. Shi S. Q. Winder D. Webb D.. (2016). Coupling optogenetic stimulation with NanoLuc-based luminescence (BRET) Ca++ sensing. Nat. Commun. 7:13268. 10.1038/ncomms13268 27786307 PMC5476805

[B104] Yeh H. W. Karmach O. Ji A. Carter D. Martins-Green M. M. Ai H. W. (2017). Red-shifted luciferase–luciferin pairs for enhanced bioluminescence imaging. Nat. Methods 14, 971–974. 10.1038/nmeth.4400 28869756 PMC5678970

[B105] Yoshida T. Kakizuka A. Imamura H. (2016). BTeam, a novel BRET-based biosensor for the accurate quantification of ATP concentration within living cells. Sci. Rep. 6:39618. 10.1038/srep39618 28000761 PMC5175186

[B106] Zhang D. Coronel-Aguilera C. P. Romero P. L. Perry L. Minocha U. Rosenfield C.. (2016). The use of a novel NanoLuc-based reporter phage for the detection of *Escherichia coli* O157: H7. Sci. Rep. 6:33235. 10.1038/srep33235 27624517 PMC5021930

[B107] Zhang J. H. Chung T. D. Oldenburg K. R. (1999). A simple statistical parameter for use in evaluation and validation of high throughput screening assays. J. Biomol. Screen. 4, 67–73. 10.1177/108705719900400206 10838414

[B108] Ziessel R. Ulrich G. Harriman A. (2007). The chemistry of Bodipy: a new El Dorado for fluorescence tools. New J. Chem. 31, 496–501. 10.1039/b617972j

